# Supplementary material for: Small-Molecule Cyclophilin Inhibitors Potently Reduce Platelet Procoagulant Activity
Source: Int J Mol Sci. 2023 Apr 12;24(8):7163. doi: 10.3390/ijms24087163 (PMC10139176; doi:10.3390/ijms24087163)
Supplement: Supplementary file 1 [file ijms-24-07163-s001.zip › Informed Consent Form_EN.pdf]

Title of the study: Blood collection from healthy volunteers for scientific research on procoagulant platelets.

Sponsor of the study: KU Leuven Campus Kulak Kortrijk, Etienne Sabbelaan 53 8500 Kortrijk

Medical Ethics Committee: UZ Leuven

Local investigator: Claudia Tersteeg, KU Leuven, 056 24 6449.

At the Laboratory for Thrombosis Research at the KU Leuven Campus Kulak Kortrijk, research projects are performed in which human blood and blood cells from healthy volunteers are used to study hemostasis. The central focus of this research is on platelets. The aim of the research is to better understand the mechanisms of hemostasis and to develop new antithrombotic strategies.

Information vital to your decision to take part

Introduction

You are being invited to take part in an interventional study. Before you agree to take part in this study, we invite you to take note of its implications in terms of organisation, possible risks and benefits, to allow you to make a decision with full awareness of the implications. This is known as giving “informed consent”.

Please read these few pages of information carefully and ask any questions you want to the investigator or his/her representative. Blood will be drawn by a doctor.

There are 2 parts to this document: the information essential to your decision, and your written consent.

If you take part in this study, you should be aware that:

- This clinical study is being conducted after having been reviewed by one or more ethics committees.
- Your participation is voluntary and must remain free from any coercion. The question to participate should not come from the hierarchical superior.
- It requires the signature of a document expressing your consent. Even after having signed this document, you can stop taking part by informing the investigator.
- The data collected on this occasion are confidential and your anonymity is guaranteed during publication of the results.
- Insurance has been taken out in case you should suffer any damage in connection with your participation in this clinical study.
- You may contact the investigator or a member of his/her team at any time should you need any additional information.

Objectives of the study

You agree to donate a small amount of blood (max. 25 mL) for research purposes at the Laboratory for Thrombosis Research. The goal of this study is to better understand primary and secondary hemostasis (blood coagulation). The focus of this research is on platelets. Different processes in which platelets play a role will be investigated in the laboratory.

Suitability for participation in the study

To be able to act as a healthy volunteer you are an adult blood donor in good health. You are over 18 years old. One week prior to the blood donation, you have not taken any medication that may affect blood clotting or platelets. This group includes, among other things, 'blood thinners', aspirin, ibuprofen, NSAIDs, ... In case of doubt, you can go to the research doctor. You are not eligible to donate blood if you have already donated at the Red Cross in the last 8 weeks.

Procedure to follow
If you agree to participate in the study, you will donate a small amount of blood (max. 25 mL). This blood sample will be processed and examined on the same day by the Laboratory for Thrombosis Research.

Rights of the participant
The blood sample that is required for the examination is only given to the researchers from the Laboratory for Thrombosis Research at the KU Leuven Campus Kulak Kortrijk after your written permission. The samples are labeled in such a way that they can't be linked to your identity. The data collected is handled without ever revealing your own identity to anyone. Your name will be known after signing this informed consent, but it will in no way be linked to the research results.

The Laboratory for Thrombosis Research remains the sole owner of the data collected and of possible discoveries derived from it. The Laboratory for Thrombosis Research formally promises to destroy all samples at the end of the storage period (after maximally 8 hours). If a medicine comes on the market after this study, you cannot derive any commercial benefit from it.

Your participation in this study is completely voluntary. If you decide not to participate, this will not be noted, and your decision will not affect the quality of care and treatment that you receive at any institution affiliated with KU Leuven.

Participation in the study
We invite you to ask any question that you think is appropriate to the responsible investigator, who will give you all the desired clarifications. The responsible doctor will also ask you to date and sign the consent form after the examination. By doing so, you confirm that you have read all of the information contained herein, that you have understood the purpose of the study, and that you have voluntarily accepted to participate.

The signed informed consent form is kept for 25 years in the archive of the Laboratory for Thrombosis Research. You will receive a copy.

Description of risks and benefits
As mentioned above, the blood collection procedures are in accordance with good medical practice. The health risks are the same as a blood test for medical purposes: a bruise, vagal syncope or experiencing very small discomfort (injection pain) at the injection site are possible.
Your participation in this study contributes to a better understanding of thrombosis and therefore to possible better treatments in the future.

Withdrawal of consent
Your participation is voluntary and you are entitled to withdraw your consent to take part in the study for any reason, without having to justify your decision.

If you take part in this study, we ask you:
- To cooperate fully in the smooth running of this study.
- Not to conceal anything such as information relating to your state of health or the medication you are taking.

Contact
If you would like additional information about this study, you can contact the following people who will be happy to help:
Prof. Dr. Claudia Tersteeg 056 24 6449

Title of the study: Blood collection from healthy volunteers for scientific research on procoagulant platelets.

II Informed consent

Participant

I declare that I have been informed of the nature of the study, its purpose, its duration, any risks and benefits and what is expected of me. I have taken note of the information document and the appendices to this document.

I have had sufficient time to think about it and discuss it with a person of my choice, such as my GP or a member of my family.

I have had the opportunity to ask any questions that came to mind and have obtained a satisfactory response to my questions.

I understand that my participation in this study is voluntary and that I am free to end my participation in this study without this affecting my relationship with the research team.

I understand that data about me will be collected throughout my participation in this study and that the investigator and the sponsor of the study will guarantee the confidentiality of these data in accordance with applicable European and Belgian legislation.

I agree to my personal data being processed as described in the section dealing with confidentiality guarantees (part III Supplementary Information).

I agree/do not agree (delete as appropriate) to the study data collected for the purposes of this study being processed at a later date provided this processing is limited to the context of the present study for a better understanding of the disease and its treatment.

I have received a copy of the information to the participant and the informed consent form.

Surname, first name, date and signature of the volunteer.

Investigator

I, the undersigned investigator, confirm that I have verbally provided the necessary information about the study and have given the participant a copy of the information document.

I confirm that no pressure was applied to persuade the patient to agree to take part in the study and that I am willing to answer any additional questions if required.

I confirm that I operate in accordance with the ethical principles set out in the latest version of the “Helsinki Declaration”, the “Good Clinical Practices” and the Belgian Law of 7 May 2004 related to experiments on humans.

Surname, first name, date and signature of the investigator

Title of the study: Blood collection from healthy volunteers for scientific research on procoagulant platelets.

1. Supplementary information on the protection and the rights of the participant in a clinical study

Ethics Committee

This study has been reviewed by an independent Ethics Committee, namely the research Ethics Committee of UZ Leuven/KU Leuven, which has issued a favourable opinion. It is the task of the Ethics Committees to protect people who take part in a clinical trial. They make sure that your rights as a patient and as a participant in a clinical study are respected, that based on current knowledge, the balance between risks and benefits remains favourable to the participants, that the study is scientifically relevant and ethical.

You should not under any circumstances take the favourable opinion of the Ethics Committee as an incentive to take part in this study.

Voluntary participation

Before signing, do not hesitate to ask any questions you feel are appropriate. Take the time to discuss matters with a trusted person if you so wish.

Your participation in the study is voluntary and must remain free of any coercion: this means that you have the right not to take part in the study or to withdraw without giving a reason, even if you previously agreed to take part. Your decision will not affect your relationship with the investigator.

If you agree to take part, you will sign the informed consent form. The investigator will also sign this form to confirm that she has provided you with the necessary information about the study. You will receive a copy of the form.

Guarantee of confidentiality

Your participation in the study means that you agree to the investigator collecting data about you and to the study sponsor using these data for research purposes and in connection with scientific and medical publications.

Your data will be processed in accordance with the European General Data Protection Regulation (GDPR) and with the Belgian legislation on the protection of natural persons with regard to the processing of personal data. KU Leuven shall act as data controller for your data. You are entitled to ask the investigator what data are being collected about you and what is their use in connection with the study.

The investigator has a duty of confidentiality vis-à-vis the data collected. This means that she undertakes not only never to reveal your name in the context of a publication or conference but also that he/she will encode (your identity will be replaced by an ID code in the study) your data before sending them to the manager of the database of collected data.

For the study data manager designated by the sponsor, the data transmitted will not allow you to be identified. The latter is responsible for collecting the data gathered by all investigators taking part in the study, processing them and protecting them in accordance with the requirements of the Belgian law on the protection of privacy.

The (encoded) study data will be able to be sent to Belgian or other regulatory authorities, to the relevant ethics committees, to other doctors and/or to organisations working in collaboration with the sponsor.

They will also be able to be sent to other sites of the sponsor in Belgium and in other countries where the standards in terms of the protection of personal data may be different or less stringent? As explained above, the transmitted data are encoded[1].

Your consent to take part in this study therefore also implies your consent to the use of your encoded medical data for the purposes described in this information form and to their transmission to the aforementioned people and authorities.

[1] The sponsor then undertakes to respect the constraints of the European General Data Protection Regulation (GDPR) and the Belgian legislation on the protection of natural persons with regard to the processing of personal data.

The sponsor undertakes only to use the data collected within the context of the study in which you are taking part.

If you have any questions about how we use your data or if you want to exercise your right to access, correct, or possibly stop further processing, you can always contact the investigator at the following contact address: Claudia Tersteeg, KU Leuven Campus Kulak Kortrijk, Etienne Sabbelaan 53, 8500 Kortrijk. If you have any special points of interest afterwards or if you wish to file a complaint, you can contact the KU Leuven privacy team at privacy@kuleuven.be.

Finally, if you have a complaint concerning the processing of your data, you can contact the Belgian supervisory authority who ensures that privacy is respected when personal data are processed.

The Belgian supervisory authority is called:
Data Protection Authority (DPA)
Drukpersstraat 35,
1000 Brussels
Tel. +32 2 274 48 00
e-mail: contact@apd-gba.be
Website: https://www.dataprotectionauthority.be

Future of your sample(s) collected during the study

A coding procedure is used for the samples. The samples submitted to the sponsor are provided with an code for this clinical study, namely "donor A, B, C, .." and the date of collection. Due to this pseudo-anonymization, there will be no direct link between the identification code and your identity and only the code will be used when processing the results.

The manager of these samples (dr. Claudia Tersteeg, Laboratory for Trombosis Research, KU Leuven Campus Kulak Kortrijk) undertakes to use them within the context of clinical research and to destroy them at the end of the scheduled storage period (= maximally 8 hours).

The sample of biological material taken is deemed to be a “donation” and you should be aware that, in principle, you will not receive any financial benefit (royalties) associated with the development of new therapies derived from the use of your donation of biological material and which may be of commercial value.

Insurance

Any participation in a clinical study involves a risk, however small it is. Even if there is no fault, the sponsor accepts responsibility for damage caused to the participant (or in the event of death, his/her dependants) and directly or indirectly linked to his/her participation in the study. The sponsor has taken out insurance for this responsibility[2].

If the investigator believes that a link with the study is possible (the insurance does not cover the natural progression of your disease or the known side effects of your normal treatment), he/she will inform the study sponsor, which will initiate the declaration procedure to the insurance company. The latter will appoint an expert - if it considers it necessary - to assess whether there is a link between your new health problems and the study.

In the event of disagreement either with the investigator or with the expert appointed by the insurance company and also whenever you feel it is appropriate, you or - in case of death - your dependants may bring proceedings against the insurer directly in Belgium (Amlin Insurance SE, contract number 299.053.700; Vanbreda Risk & Benefits NV, Plantin en Moretuslei 297, 2140 Antwerpen).

The law provides that the insurer may be summoned to appear either before the judge of the location where the event giving rise to the damage occurred, or before the judge of your domicile, or before the judge of the insurer’s registered offices.

[2] In accordance with Article 29 of the Belgian Law related to experiments on humans (7 May 2004)
