# Supplementary material for: Small-Molecule Cyclophilin Inhibitors Potently Reduce Platelet Procoagulant Activity
Source: Int J Mol Sci. 2023 Apr 12;24(8):7163. doi: 10.3390/ijms24087163 (PMC10139176; doi:10.3390/ijms24087163)
Supplement: Supplementary file 1 [file ijms-24-07163-s001.zip › Supplementals_text.pdf]

Supplementals of "Small-molecule cyclophilin inhibitors potently reduce platelet procoagulant activity"

Jens Van Bael [1], Aline Vandenbulcke [1], Abdelhakim Ahmed-Belkacem [2], Jean-François Guichou [3], Jean-Michel Pawlotsky [2,4], Jelle Samyn [1], Arjan D. Barendrecht [5], Coen Maas [5], Simon F. De Meyer [1], Karen Vanhoorelbeke [1], Claudia Tersteeg [1,*]

[1] Laboratory for Thrombosis Research, KU Leuven Campus Kulak, Kortrijk, Belgium
[2] INSERM U955, Team Viruses, Hepatology Cancer, Créteil, France
[3] Centre de Biologie Structurale (CBS), INSERM U1054, CNRS UMR5048, Université de Montpellier, Montpellier, France
[4] National Reference Center for Viral Hepatitis B, C and Delta, Department of Virology, Hôpital Henri Mondor, Université Paris-Est, Créteil, France
[5] Diagnostic Laboratory Research, UMC Utrecht, Utrecht, The Netherlands
* Correspondence: claudia.tersteeg@kuleuven.be; Tel.: +3256246449

Supplemental Figure A1: SMCypIs do not affect platelet activation. Washed platelets were incubated with 125 µM F759 or F83236, or 4 µM CsA. Platelets were then activated with either ADP (1 or 10 µM), CRP-XL (0.1 or 1 µg/mL) or thrombin (0.2 or 0.8 U/mL). During activation, platelets were labeled with anti-CD42b, anti-CD62P and anti-activated $\alpha_{IIb}\beta_3$ (PAC-1 clone). $CD42b^+/CD62P^+$/activated $\alpha_{IIb}\beta_3^+$ platelets were analyzed using flow cytometry. **(a)** Percentages of $CD42b^+/CD26P^+$ platelets. **(b)** Percentages of $CD42b^+$/activated $\alpha_{IIb}\beta_3^+$ platelets. Data are represented as mean ± SD of 4 separate experiments. SMCypIs, small-molecule cyclophilin inhibitors; CsA, Cyclosporin A; ADP, adenosine 5'-diphosphate; CRP-XL, collagen related peptide.

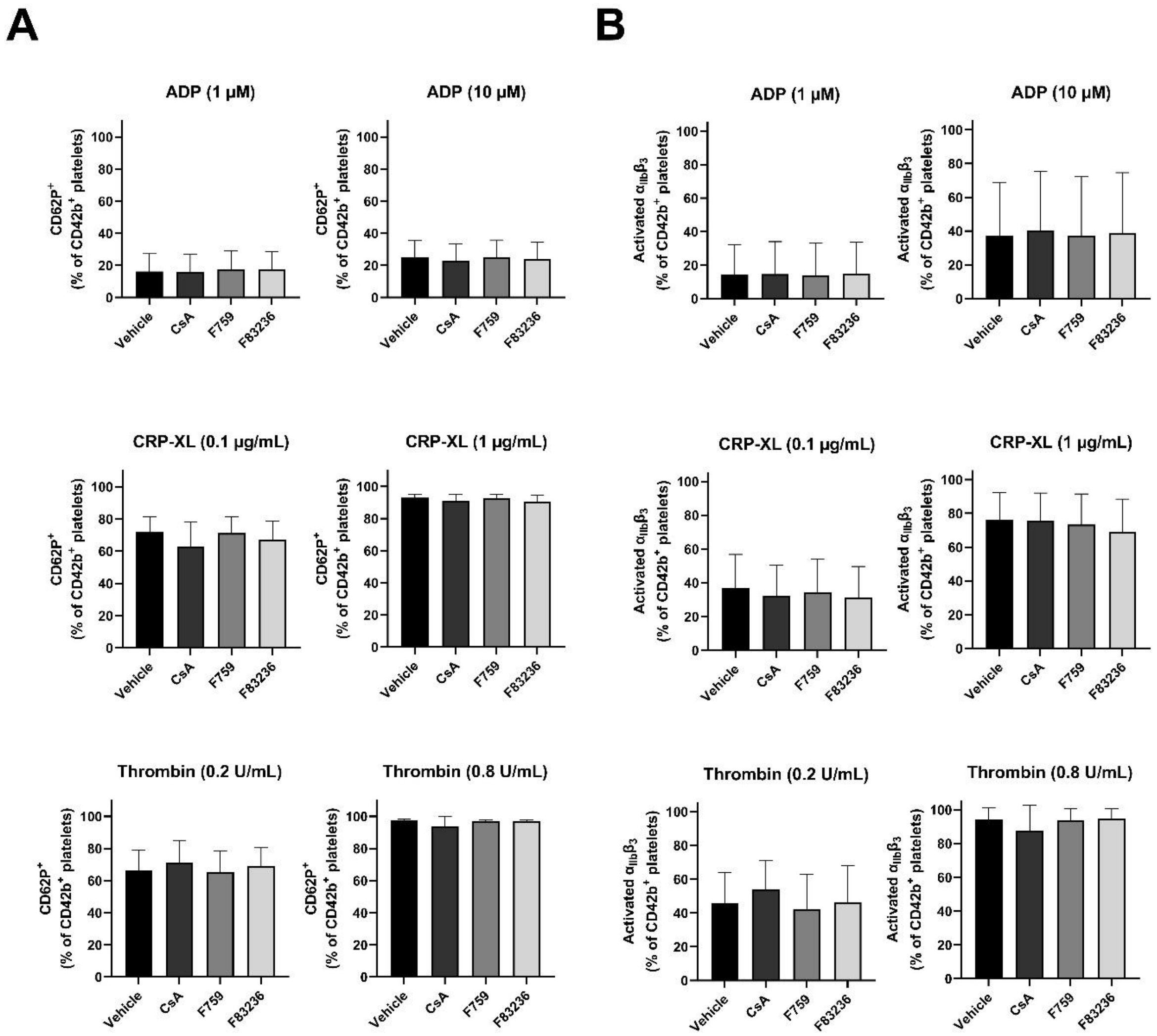

Supplemental Figure A2: Chemical structures of the SMCypIs and CsA

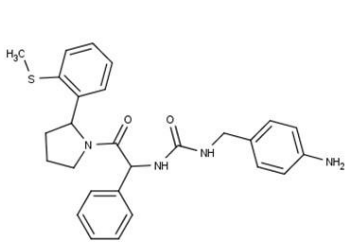

F759

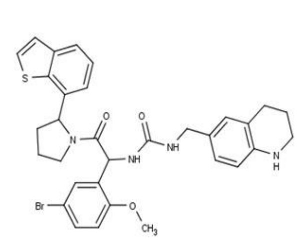

F83236

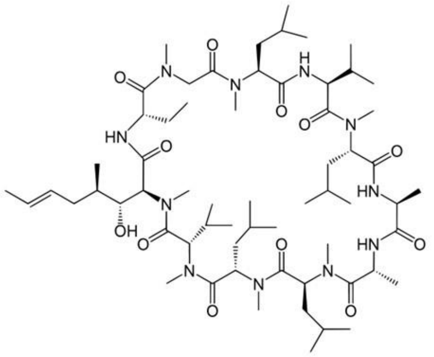

Cyclosporine A

Supplemental Video A3: SMCypIs inhibit coagulation induced by procoagulant platelets. Whole blood was incubated with 125 μM F759 or F83236, 4 μM CsA before perfusion over a Horm collagen coated surface, in the presence of 6.6 mM $CaCl_2$ and 3.08 $MgCl_2$. Shown are representative videos of: (i) top row: DIC images of platelet aggregates; (ii) middle row: anti-GpIb-VhH Alexa647 indicating platelets and (iii) bottom row: tPA-Alexa488 binding to fibrin. SMCypIs, small-molecule cyclophilin inhibitors; CsA, Cyclosporin A; tPA, tissue plasminogen activator.

Supplemental Data A4: Informed consent form.

Informed consent form of clinical study (S64328) to obtain blood from healthy individuals.
